# Enhanced Recovery Independently Lowers Failure to Rescue After Colorectal Surgery

**DOI:** 10.1097/DCR.0000000000003655

**Published:** 2025-02-11

**Authors:** Marco Catarci, Giacomo Ruffo, Massimo Giuseppe Viola, Gianluca Garulli, Maurizio Pavanello, Marco Scatizzi, Vincenzo Bottino, Stefano Guadagni

**Affiliations:** 1 General Surgery Unit, Sandro Pertini Hospital, ASL Roma 2, Roma, Italy; 2 General Surgery Unit, IRCCS Sacro Cuore Don Calabria Hospital, Negrar di Valpolicella (VR), Italy; 3 General Surgery Unit, Cardinale G. Panico Hospital, Tricase (LE), Italy; 4 General Surgery Unit, Infermi Hospital, Rimini, Italy; 5 General Surgery Unit, AULSS2 Marca Trevigiana, Conegliano Veneto (TV), Italy; 6 General Surgery Unit, Santa Maria Annunziata and Serristori Hospital, Florence, Italy; 7 General and Oncologic Surgery Unit, Evangelico Betania Hospital, Napoli, Italy; 8 General Surgery Unit, Università degli Studi dell’Aquila, L’Aquila, Italy

**Keywords:** Colorectal surgery, Enhanced recovery after surgery, Failure to rescue

## Abstract

**BACKGROUND:**

High adherence to the enhanced recovery after surgery pathway reduces morbidity and mortality rates after elective colorectal surgery.

**OBJECTIVE:**

To evaluate the effect of adherence to the enhanced recovery after surgery pathway on the failure to rescue rates after elective colorectal surgery.

**DESIGN:**

Retrospective analysis of a prospective database.

**PATIENTS:**

Adults (18 years or older) who underwent elective colorectal resection with anastomosis for benign and malignant disease.

**SETTINGS:**

Prospective enrollment in 78 centers in Italy from 2019 to 2021.

**INTERVENTIONS:**

All outcomes were measured 60 days after surgery. Several patient-, disease-, treatment-, hospital-, and complication-related variables were analyzed. After univariate analyses, independent predictors of the end points were identified through logistic regression analyses, presenting ORs and 95% CIs.

**MAIN OUTCOME MEASURES:**

Failure to rescue after any adverse event, defined as the ratio between the number of deaths and the number of patients showing any adverse event; failure to rescue after any major adverse event, with the denominator represented by the number of patients showing any major adverse event.

**RESULTS:**

An adverse event was recorded in 2321 of 8359 patients (27.8%), a major adverse event in 523 patients (6.3%), and death in 88 patients (1.0%). The failure to rescue rates were 3.8% after any adverse event and 16.8% after any major adverse event. Independent predictors of primary end points were identified among patient- (age, ASA class, and nutritional status), treatment- (type of resection), and complication-related (anastomotic leakage and reoperation) variables. Enhanced recovery pathway adherence of more than 70% independently reduced failure to rescue rates.

**LIMITATIONS:**

Clustering from multicenter data and unmeasured confounding from observational data.

**CONCLUSIONS:**

After elective colorectal resection, adherence of more than 70% to the enhanced recovery pathway independently decreased failure to rescue rates, along with other patient- or treatment-related factors. See **Video Abstract**.

**LA RECUPERACIÓN MEJORADA REDUCE DE FORMA INDEPENDIENTE LA POSIBILIDAD DE FRACASO EN EL RESCATE DESPUÉS DE UNA CIRUGÍA COLORRECTAL:**

**ANTECEDENTES:**

La alta adherencia a la vía de recuperación mejorada después de la cirugía reduce las tasas de morbilidad y mortalidad después de la cirugía colorrectal electiva.

**OBJETIVO:**

Evaluar el efecto de la adherencia a la vía ERAS en las tasas de fracaso en el rescate después de la cirugía colorrectal electiva.

**DISEÑO:**

Análisis retrospectivo de una base de datos prospectiva.

**PACIENTES:**

Adultos (≥ 18 años) que se sometieron a una resección colorrectal electiva con anastomosis por enfermedad benigna y maligna.

**ESCENARIO:**

Inscripción prospectiva en 78 centros en Italia de 2019 a 2021.

**INTERVENCIONES:**

Todos los resultados se midieron a los 60 días después de la cirugía. Se analizaron varias variables relacionadas con el paciente, la enfermedad, el tratamiento, el hospital y las complicaciones para los resultados. Después de los análisis univariados, se identificaron los predictores independientes de los puntos finales a través de análisis de regresión logística, presentando razones de probabilidades e intervalos de confianza del 95%.

**PRINCIPALES MEDIDAS DE RESULTADOS:**

Fallo en el rescate después de cualquier evento adverso, definido como la relación entre el número de muertes y el número de pacientes que presentaron cualquier evento adverso; fallo en el rescate después de cualquier evento adverso mayor, con el denominador representado por el número de pacientes que presentaron cualquier evento adverso mayor.

**RESULTADOS:**

Se registró un evento adverso en 2321 de 8359 pacientes (27,8%), un evento adverso mayor en 523 pacientes (6,3%) y muerte en 88 pacientes (1,0%). Las tasas de fallo en el rescate fueron del 3,8% después de cualquier evento adverso y del 16,8% después de cualquier evento adverso mayor. Se identificaron predictores independientes de los criterios de valoración primarios entre las variables relacionadas con el paciente (edad, clase de la Sociedad Americana de Anestesiólogos, estado nutricional), el tratamiento (tipo de resección) y las complicaciones (fuga anastomótica, reoperación). La adherencia a la vía de recuperación mejorada > 70% redujo de forma independiente las tasas de fallo en el rescate.

**LIMITACIONES:**

Agrupamiento de datos multicéntricos y factores de confusión no medidos a partir de datos observacionales.

**CONCLUSIONES:**

Después de una resección colorrectal electiva, la adherencia > 70 % a la vía de recuperación mejorada disminuyó de manera independiente las tasas de fracaso en el rescate, junto con otros factores relacionados con el paciente o el tratamiento. *(Traducción—Dr Osvaldo Gauto).*

Failure to rescue (FTR) was introduced more than 30 years ago as a new metric to describe the rate of death among patients with any postoperative complication,^1^ capturing the spiral of patients’ deterioration from an initial complication to subsequent complications and death. It rapidly became an internationally accepted quality indicator,^2,3^ but its original definition was modified many times in changing the ratio denominator, thus making comparisons between studies very difficult if possible at all.^4^ Regarding colorectal surgery (CRS), the rates of FTR after major complications (FTR-MC), or the ratio between the number of deaths and the number of patients experiencing any major complication, were significantly lower in academic hospitals,^5^ in centers with the highest availability of intensive care beds,^6^ and urban hospitals^7^; they were not influenced by hospital volume^8^ or teaching status.^9^ Conversely, hospital volume was reported to be the strongest predictor of complications and FTR after rectal surgery in Italy.^10^

The rates of FTR after reoperations, or the ratio between the number of deaths and the number of patients undergoing unplanned reoperations, were significantly higher in centers with the highest mortality rates.^11^ In contrast, a recent nationwide study in New Zealand^12^ showed that the primary driver of the recorded reduction in mortality rates after CRS during the past few years was the reduction of FTR after nonoperative management of nonsurgical (ie, cardiac, pulmonary, renal, and neurological) complications.

High adherence to the enhanced recovery after surgery (ERAS) pathway is linked to a significant and independent reduction of overall morbidity (OM), major morbidity (MM), and mortality rates after CRS.^13–17^ Whether this effect is mediated by a reduction in FTR rates has not yet been investigated. Therefore, the Italian ColoRectal Anastomotic Leakage (iCral) study group decided to investigate the impact of ERAS pathway adherence on FTR rates after CRS using the results of 2 prospective studies designed to analyze other end points.^18,19^

## MATERIALS AND METHODS

**FIGURE 1.**
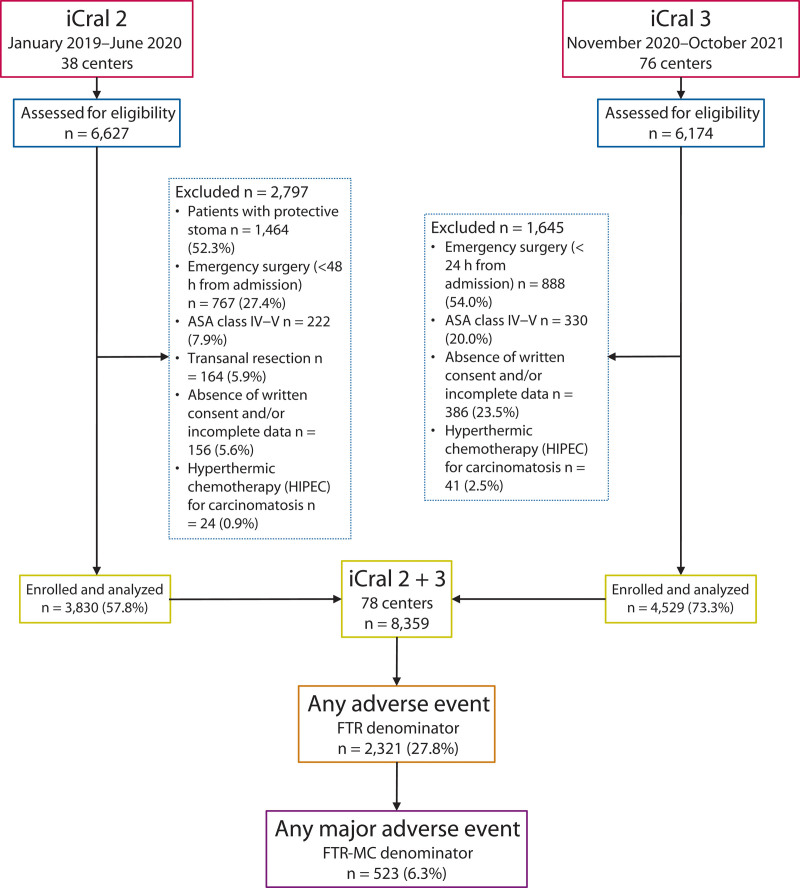
Flow chart of the iCral2^18^ and iCral3^19^ studies according to the Strengthening the Reporting of Observational Studies in Epidemiology guidelines.^20^ FTR = failure to rescue after any complication; FTR-MC = failure to rescue after major complications; HIPEC = hyperthermic chemotherapy; iCral = Italian ColoRectal Anastomotic Leakage study group; STROBE = Strengthening the Reporting of Observational Studies in Epidemiology.

This was a planned retrospective analysis of a database based on prospective enrollment on a voluntary basis, from January 2019 to September 2021, in 78 surgical centers in Italy (iCral2 and iCral3 studies).^18,19^ According to explicit inclusion/exclusion criteria that were similar for the 2 studies, all patients who had elective CRS with anastomosis were evaluated for inclusion (see Supplemental Table 1 at http://links.lww.com/DCR/C460). Patients who had a protective stoma proximal to the anastomosis were not included in the iCral2 study but were included in the iCral3 study. Delayed urgency resections were defined as occurring >48 hours after admission in the iCral2 study and >24 hours after admission in the iCral3 study (Fig. 1). Sample sizes were computed and presented separately.^18,19^ The Strengthening the Reporting of Observational Studies in Epidemiology reporting criteria for cohort studies were adhered to in both investigations.^20^ Each participating center was defined as high volume (4 or more cases) or low volume (fewer than 4 cases) according to the median number of enrolled cases per month, as metropolitan/academic or local/rural hospital, and as general or colorectal/oncologic surgery unit. ERAS pathway was defined on 20 items recorded in the 2 studies, derived from international and national guidelines,^21,22^ with adherence measured through explicit criteria (see Supplemental Table 2 at http://links.lww.com/DCR/C460).

Through an electronic case report form secured by access credentials for each center or investigator, all of the enrolled patients’ data were prospectively entered into a web-based database. Both continuous and discrete variables pertaining to biometric information, risk factors associated with the patient, surgical procedure type and indication, ERAS pathway item adherence, and results were documented. Each record was subjected to quality control by local investigators for consistency, plausibility, and completeness. The study coordinator then verified the data, resolving any discrepancies through rigorous collaboration. During the perioperative period, patients were examined daily by local investigators, who were free to decide on complementary imaging and any further action according to local criteria.

### Outcomes

All outcomes were calculated at 60 days after surgery. Any adverse event was recorded, classified, and graded according to Clavien-Dindo^23^ and the Japanese Clinical Oncology Group extended criteria,^24^ as well as any reoperation and any death. Anastomotic leakage (AL) was defined according to international consensus.^25^ OM was defined as any adverse event, whereas MM as any major adverse event (Clavien-Dindo grade higher than II).

The end points were FTR, defined as the ratio between the number of deaths and the number of patients experiencing any complication, and FTR after major complications (FTR-MC), defined as the ratio between the number of deaths and the number of patients experiencing any major complication.

### Statistical Analysis

All quantitative values were expressed as mean ± SD and 95% CI, categorical data as percentage frequencies, and discrete variables as median and interquartile range (IQR).

Age (years), BMI, and operation duration (minutes) were all classified as being below/at or above their median values. Adherence to the ERAS pathway was categorized either below/at or above its median value or according to its quartiles. The surgical approach was categorized either as open vs minimally invasive (laparoscopic, robotic, and converted) or according to the 4 types. The mini nutritional assessment–short form (MNA-SF)^26^ was used for nutritional screening, and results were classified as being below or at 11 or above 11, which represents the normal nutrition status threshold. Anterior resection, right colectomy, and left colectomy were classified as standard surgical procedures, whereas splenic flexure resection, transverse colectomy, Hartmann’s reversal, subtotal and total colectomy, and other resections were classified as nonstandard, the former having an independent lower impact on postoperative complications.^18^ Any anastomosis located within 10 cm from the external anal verge identified a rectal resection, and all the others were classified as colon resections.

Univariable analyses of the study cohorts according to the end points were performed using cross-tabulations with the χ^2^ test and/or Fisher exact test. A multiple linear regression model measuring the variance inflation factor was used to test for multicollinearity^27^ of all variables exhibiting significance at univariate analysis; any variable showing a variance inflation factor of more than 4 was further tested with pairwise comparisons and excluded in case of significant collinearity with other variables, whereas the others were included in a multivariate analysis model for the end points using logistic regression, showing OR and 95% CI.

The significance level for every statistical test was set at a *p* value of <0.05. StatsDirect statistical software (version 1.9.8; StatsDirect Ltd, United Kingdom) was used for all analyses.

### Ethics

The Declaration of Helsinki and the principles of the E6 (*R*2) guidelines for good clinical practice guided both studies. After approval by the coordinating center ethics committee (Marche Regional Ethics Committee 2018/334 released on November 28, 2018, for iCral2; 2020/192 released on July 30, 2020, for iCral3) and the local ethics committee of all the other participating centers, the study protocols were registered at ClinicalTrials.gov (NCT03771456 for iCral2 and NCT04397627 for iCral3). For both studies, anonymized data sets at the individual participant level are available on specific request to the study coordinator.

## RESULTS

The 2 studies enrolled a total of 8359 patients (Fig. 1), with a median (IQR; range) of 62 (42–140; 12–674) per single center. The median (IQR; range) follow-up was 65 days (55–100; 0–378). A total of 3171 adverse events were recorded in 2321 patients (OM rate 27.8%), representing the population at the FTR denominator. Details about the type and grade of any adverse event were previously reported.^17–19^ There were 774 (24.2%) major adverse events (Table 1) in 523 patients (MM rate 6.3%), representing the population at the FTR-MC denominator. The numerator of both FTR and FTR-MC was represented by 88 deaths (1.0%). The FTR and FTR-MC rates were 3.8%. and 16.8%, respectively. There were 366 ALs (4.4%) and 429 reoperations (5.1%).

**TABLE 1.** Major adverse events and grading in 523 patients

Adverse events	*Clavien-Dindo*^*23*^ *and JCOG-PC*^*24*^ *grade*				
	*IIIa*	*IIIb*	*IVa*	*IVb*	*Total*
Anastomotic leakage	34	218	30	12	294
Incisional surgical site infections (superficial and deep)	16	17	0	0	33
Organ/space surgical site infections (abdominal collection/abscess)	53	15	0	2	70
Small bowel obstruction	4	62	2	0	68
Anastomotic bleeding	40	3	1	0	44
Abdominal bleeding	7	36	4	2	49
Small bowel perforation	1	19	2	0	22
Trocar/wound site bleeding	2	3	0	0	5
Acute mesenteric ischemia	0	4	0	1	5
Acute peptic ulcer/gastritis	4	1	0	0	5
Anemia	1	2	0	1	4
Cardiac dysfunction and failure	30	3	8	10	51
Fever	3	3	0	0	6
DVT/pulmonary embolism	1	0	3	5	9
Neurologic	1	1	0	1	3
Pneumonia and pulmonary failure	8	2	21	9	40
Urinary tract infection	1	0	0	0	1
Acute renal failure	3	0	6	2	11
Other	0	21	1	2	24
Total	213	412	91	58	744

DVT = deep venous thrombosis; JCOG-PC = Japan Clinical Oncology Group postoperative complications.

**FIGURE 2.**
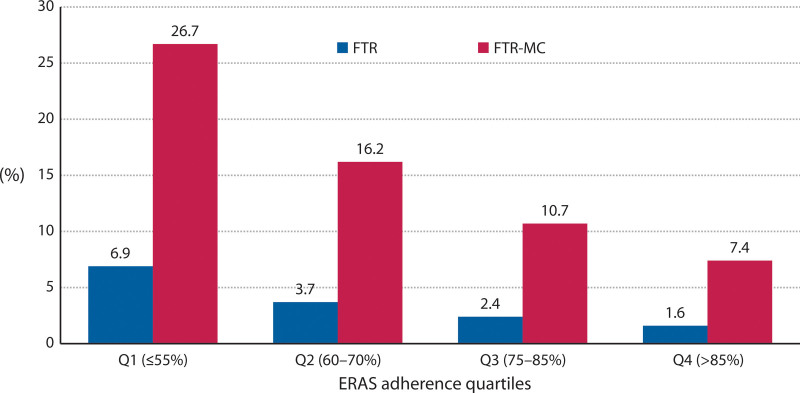
Trend of the end points according to the ERAS adherence quartiles (Q1–Q4); *p* < 0.0001, the χ^2^ test *df* = 3. ERAS = enhanced recovery after surgery; FTR-MC = failure to rescue after major complications.

After multivariate analysis, FTR rates (Table 2) were independently higher by age older than 69 years vs 69 years or younger (6.9% vs 1.2%; OR 2.66; 95% CI, 1.44–4.92; *p* = 0.002), ASA class III vs I and II (6.5% vs 1.9%; OR 1.88; 95% CI, 1.13–3.15; *p* = 0.015), and AL (7.1% vs 3.2%; OR 2.33; 95% CI, 1.42 to 3.81; *p* < 0.001). They were independently lower by MNA-SF of more than 11 vs 11 or less (2.4% vs 6.6%; OR 0.47; 95% CI, 0.30–0.74; *p* = 0.001), and ERAS pathway adherence of more than 70% vs 70% or less (2.0% vs 5.3%; OR 0.45; 95% CI, 0.27–0.76; *p* = 0.003). FTR-MC rates (Table 3) were independently higher by age older than 69 years vs 69 years or younger (24.9% vs 6.5%; OR 2.42; 95% CI, 1.16–5.04; *p* = 0.018), and ASA class III vs I and II (27.3% vs 8.8%; OR 2.08; 95% CI, 1.09–3.98; *p* = 0.026). They were independently lower by MNA-SF of more than 11 vs 11 or less (11.0% vs 28.4%; OR 0.43; 95% CI, 0.24–0.79; *p* = 0.006), standard vs nonstandard resection (15.1% vs 27.4%; OR 0.30; 95% CI, 0.14–0.65; *p* = 0.002), ERAS pathway adherence of more than 70% vs 70% or less (9.1% vs 22.9%; OR 0.41; 95% CI, 0.21–0.80; *p* = 0.009), AL (9.0% vs 26.4%; OR 0.50; 95% CI, 0.26–0.94; *p* = 0.031), and reoperation (7.5% vs 59.6%; OR 0.11; 95% CI, 0.06–0.20; *p* < 0.0001). Moreover, the effect of ERAS adherence rates on both end points was consistent across its quartiles (Fig. 2).

**TABLE 2.** Logistic regression analysis for FTR in 2321 patients with any complication

			*Univariate analysis*				*Multivariate analysis* ^a^				
*Variable and pattern*	*N*	*%*	*Events*	*%*	*p*	*VIF*	*Beta*	*Beta SE*	*OR*	*95% CI*	*p*
Age, y											
≤69	1268	54.6	15	1.2							
>69	1053	45.4	73	6.9	<0.0001	1.2687	0.9783	3.1129	2.66	1.44–4.92	0.002
BMI											
≤25.13	1180	50.8	45	3.8	0.95						
>25.13	1141	49.2	43	3.8							
Sex											
Female	1075	46.3	32	3.0	0.056						
Male	1246	53.7	56	4.5							
ASA class											
I–II	1363	58.7	26	1.9							
III	958	41.3	62	6.5	<0.0001	1.2524	0.6338	2.4215	1.88	1.13–3.15	0.015
MNA-SF											
≤11	755	32.5	50	6.6							
>11	1566	67.5	38	2.4	<0.0001	1.0456	–0.7467	–3.2538	0.47	0.30–0.74	0.001
Preoperative immune enhancing nutrition											
No	1615	69.6	69	4.3	0.06						
Yes	706	30.4	19	2.7							
Diabetes											
No	1948	83.9	72	3.7	0.58						
Yes	373	16.1	16	4.3							
Chronic renal failure											
No	2214	95.4	79	3.6							
Yes	107	4.6	9	8.4	0.018	1.0168	0.5769	1.5050	1.78	0.84–3.77	0.13
Neoadjuvant therapy											
No	2175	93.7	81	3.7	0.85						
Yes	176	7.6	7	4.0							
Chronic liver disease											
No	2292	98.8	85	3.7	0.12						
Yes	29	1.2	3	10.3							
Center volume											
<4 cases/mo	659	28.4	22	3.3	0.47						
≥4 cases/mo	1662	71.6	66	4.0							
Hospital type											
Academic/metropolitan	1726	74.4	70	4.1	0.25						
Local/rural	595	25.6	18	3.0							
Unit type											
General	1965	84.7	73	3.7	0.65						
Colorectal/oncologic	356	15.3	15	4.2							
Preoperative blood transfusions											
No	2155	92.8	75	3.5							
Yes	166	7.2	13	7.8	0.005	1.0514	0.3067	0.9306	1.36	0.71–2.59	0.35
Intra- and postoperative blood transfusions											
No	1946	83.8	70	3.6	0.26						
Yes	375	16.2	18	4.8							
Surgery for malignancy											
No	603	26.0	13	2.2							
Yes	1718	74.0	75	4.4	0.014	1.1382	0.0834	0.2581	1.09	0.58–2.05	0.79
Standard procedure											
Yes	1918	82.6	68	3.5	0.17						
No	403	17.4	20	5.0							
Type of resection											
Colon	1668	71.9	68	4.1	0.25						
Rectum	653	28.1	20	3.1							
Operation length, min											
≤180	1261	54.3	47	3.7	0.86						
>180	1060	45.7	41	3.9							
Minimally invasive surgery											
No	422	18.2	31	7.3							
Yes	1899	81.8	57	3.0	<0.0001	2.0478	–0.4884	–1.9603	0.61	0.38–1.00	0.05
Surgical approach											
Open	422	18.2	31	7.3	<0.0001	4.1254	Excluded				
Laparoscopic	1519	65.4	34	2.2							
Robotic	209	9.0	13	6.2							
Converted	171	7.4	10	5.8							
ERAS adherence rate, %											
≤70	1258	54.2	67	5.3							
>70	1063	45.8	21	2.0	<0.0001	2.1526	–0.7957	–2.9595	0.45	0.27–0.76	0.003
ERAS adherence quartiles, %											
Q1 (≤55)	641	27.6	44	6.9	<0.0001	4.2946	Excluded				
Q2 (60–70)	617	26.6	23	3.7							
Q3 (75–85)	508	21.9	12	2.4							
Q4 (>85)	555	23.9	9	1.6							
Major morbidity											
No	1798	77.5	0	0.0							
Yes	523	22.5	88	16.8	<0.0001	4.0782	Excluded				
Anastomotic leakage											
No	1955	84.2	62	3.2							
Yes	366	15.8	26	7.1	<0.0003	1.6194	0.8452	3.3676	2.33	1.42–3.81	<0.001
Reoperation											
No	1892	81.5	56	3.0							
Yes	429	18.5	32	7.5	<0.0001	4.2112	Excluded				

beta = regression coefficient; ERAS = enhanced recovery after surgery; FTR = failure to rescue; MNA-SF = mini nutritional assessment short form^26^; Q1–Q4 = quartiles of ERAS adherence; VIF = variation inflation factor.

a Deviance (likelihood ratio) χ^2^ = 93.62, *df* = 9, *p* < 0.0001.

**TABLE 3.** Logistic regression analysis for failure to rescue in 523 patients with any major complication

			*Univariate analysis*				*Multivariate analysis*				
*Pattern*	*N*	*%*	*Events*	*%*	*p*	*VIF*	*Beta*	*Beta SE*	*OR*	*95% CI*	*p*
Age, y											
≤69	230	44.0	15	6.5							
>69	293	56.0	73	24.9	<0.0001	1.3722	0.8837	2.3563	2.42	1.16–5.04	0.018
BMI											
≤25.13	269	51.4	45	16.7	0.951						
>25.13	254	48.6	43	16.9							
Sex											
Female	232	44.4	32	13.8	0.098						
Male	291	55.6	56	19.2							
ASA class											
I–II	296	56.6	26	8.8							
III	227	43.4	62	27.3	<0.0001	1.3515	0.7340	2.2194	2.08	1.09–3.98	0.026
MNA-SF											
≤11	176	33.7	50	28.4	<0.0001						
>11	347	66.3	38	11.0		1.0623	–0.8313	–2.7296	0.43	0.24–0.79	0.006
Preoperative immune-enhancing nutrition											
No	367	70.2	69	18.8	0.064						
Yes	156	29.8	19	12.2							
Diabetes											
No	438	83.7	72	16.4	0.591						
Yes	85	16.3	16	18.8							
Chronic renal failure											
No	491	93.9	79	16.1	0.088						
Yes	32	6.1	9	28.1							
Neoadjuvant therapy											
No	475	90.8	81	17.1	0.839						
Yes	48	9.2	7	14.6							
Chronic liver disease											
No	519	99.2	85	16.4	0.016						
Yes	4	0.8	3	75.0		1.0326	1.5894	1.0346	4.90	0.24–99.51	0.301
Center volume											
<4 cases/mo	131	25.0	22	16.8	0.991						
≥4 cases/mo	392	75.0	66	16.8							
Hospital type											
Academic/metropolitan	415	79.3	70	16.9	0.96						
Local/rural	108	20.7	18	16.7							
Unit type											
General	429	82.0	73	17.0	0.804						
Colorectal/oncologic	94	18.0	15	16.0							
Preoperative blood transfusions											
No	485	92.7	75	15.5							
Yes	38	7.3	13	34.2	0.003	1.0445	0.7054	1.5085	2.02	0.81–5.06	0.131
Intra- and postoperative blood transfusions											
No	428	81.8	70	16.4	0.541						
Yes	95	18.2	18	18.9							
Surgery for malignancy											
No	114	21.8	13	11.4							
Yes	409	78.2	75	18.3	0.08						
Standard procedure											
Yes	73	14.0	20	27.4	0.009						
No	450	86.0	68	15.1		1.0447	–1.1925	–3.0829	0.30	0.14–0.65	0.002
Type of resection											
Colon	355	67.9	68	19.1							
Rectum	168	32.1	20	11.9	0.038	1.1031	–0.3173	–0.9020	0.73	0.36–1.45	0.367
Operation length, min											
≤180	317	60.6	52	16.4	0.749						
>180	206	39.4	36	17.5							
Minimally invasive surgery											
No	97	18.5	31	32.0							
Yes	426	81.5	57	13.4	<0.0001	2.2001	–0.2766	–0.7843	0.76	0.38–1.51	0.433
Surgical approach											
Open	97	18.5	31	32.0	<0.0001	4.1215	Excluded				
Laparoscopic	351	67.1	34	9.7							
Robotic	43	8.2	13	30.2							
Converted	32	6.2	10	31.2							
ERAS adherence rate, %											
≤70	293	56.0	67	22.9							
>70	230	44.0	21	9.1	<0.0001	2.1476	–0.8853	–2.6004	0.41	0.21–0.80	0.009
ERAS adherence quartiles, %											
Q1 (≤55)	176	33.6	47	26.7	<0.0001	4.2372	Excluded				
Q2 (60–70)	117	22.4	19	16.2							
Q3 (75–85)	149	28.5	16	10.7							
Q4 (> 85)	81	15.5	6	7.4							
Anastomotic leakage											
No	229	43.8	60	26.2							
Yes	294	56.2	28	9.5	<0.0001	1.1881	–0.6997	–2.1625	0.50	0.26–0.94	0.031
Reoperation											
No	94	19.5	56	59.6							
Yes	429	80.5	32	7.5	<0.0001	1.2065	–2.2379	–6.9162	0.11	0.06–0.20	<0.0001

Deviance (likelihood ratio) χ^2^ = 170.98, *df* = 11, *p* < 0.0001.

beta = regression coefficient; ERAS = enhanced recovery after surgery; MNA-SF = mini nutritional assessment short form; Q1–Q4 = quartiles of ERAS adherence; VIF = variation inflation factor.

## DISCUSSION

To the best of our knowledge, the present study is the first to investigate the relationship between adherence to the ERAS pathway and FTR rates after CRS. ERAS is a multidisciplinary evidence-based approach for optimizing patient recovery after surgery. This includes the use of pre-, intra-, and postoperative protocols aimed at reducing pain, speeding recovery, and minimizing complications. In contrast, FTR refers to the ability of a health care system to timely recognize and properly manage complications that may occur after surgery. This study clearly showed that satisfactory (ie, more than 70%) compliance with the ERAS pathway somehow improved the ability of the health care system in this study to effectively manage any complications that occurred after CRS, independent of other factors. This finding reinforces the need to homogenize ERAS implementation across surgical units.^28^

It is quite straightforward that patient-related risk factors such as older age, multiple comorbidities, and subnormal nutritional status play a major role as risk factors for both FTR and FTR-MC, as previously reported^2,6,7,12^; however, they cannot entirely explain FTR, which also depends on technical measures leading to early warning/diagnosis of complications and organizational measures of the team/hospital designed for their optimal management.^29^ In contrast, AL showed a controversial significant influence on FTR and FTR-MC rates; in patients experiencing any adverse event (Table 2), the absence of AL was related to a significant decrease in the risk of mortality (OR 0.25); conversely, its presence was a protective factor (OR 0.47) in patients experiencing any major adverse event (Table 3), mostly mediated by reoperation (OR 0.11). This apparently controversial finding depends on 2 considerations: 1) since its inception, the iCral study group focused on early diagnosis and management of AL after CRS, demonstrating that this focus determined an improvement of early and late results,^30,31^ and 2) nearly 80% of major adverse events in this study were represented by “surgical” complications (Table 1), which were amenable to resolution through a reoperation. Two recent nationwide studies, after CRS in New Zealand^12^ and after major abdominal surgery in Norway,^32^ showed that variations in FTR rates across different centers and periods mostly depend on the diagnosis and management of nonsurgical (ie, cardiac, pulmonary, renal, and neurological) complications. Therefore, early identification of high-risk patients with nonsurgical complications for perioperative management by a multidisciplinary (medical and surgical) team could be of paramount importance^33^ in reducing FTR rates.

It should be noted that the occurrence of any major complication had a strong influence on FTR rates in the entire population (Table 2), with all deaths recorded in patients with at least 1 major complication. This finding mostly depends on 2 factors: 1) the confirmation that FTR is able to capture the spiral of patients’ deterioration from any initial adverse event to further major adverse events and death^1,4^; and 2) the fact that any complication recorded during the 60-day postoperative follow-up in this study was considered to be surgery-related.

Minimally invasive surgery, performed in more than 80% of cases in the present study, was significant for both FTR and FTR-MC after univariate analyses but lost significance in multivariate analyses, confirming it to be just a component of the ERAS pathway, as suggested more than a decade ago.^34^

No significant influence of center caseload on either end point was recorded in the present analysis, reflecting the controversial results reported to date. Although an annual threshold of 30 colectomies or 20 proctectomies per center (roughly corresponding to the median value considered in the present analysis) lowered FTR rates by 30% in a large retrospective study of the German Cancer Society,^35^ hospital volume thresholds did not appear to have any influence on FTR rates in the Swedish Colorectal Cancer Registry.^8^ Although a greater caseload is generally reported by larger, metropolitan, and teaching hospitals,^5–10^ it is possible that micro-system organizational aspects (intensive care bed availability, nurse-to-patient ratio, early warning response teams) may be present as well in minor, local and nonteaching hospitals. Limitations of the present study that prevent any further interpretation of this finding are discussed below.

This study inexorably incorporates the strengths and limitations of its parent studies.^18,19^ Its main strength is a large number of enrolled patients in a well-defined time lapse, which makes it one of the biggest prospective cohorts to examine the impact of ERAS pathway adherence, measured through clear and sheer compliance criteria, on outcomes after CRS. Moreover, the large number of participating centers represents a wide sample of surgical units that perform colorectal resections in Italy. Although several hospital-related variables (hospital volume, hospital type, and unit type) were included in the analyses, the main limitation is the multicenter nature of the data (coming from 78 different centers in Italy), which may entail a clustering bias.^36^ Another limitation is linked to the exclusion criteria adopted, with the results not being generalizable to the excluded population (ie, ASA class IV, emergent procedures). Moreover, several factors with potential impact on FTR rates were not accounted for in the parent studies: nurse-to-patient ratio, hospital size, intensive care bed availability, hospital early warning, and response teams. Finally, any observational study has inherent limitations, including the possibility of residual confounding factors.

## CONCLUSIONS

The results of this study showed that compliance with the ERAS pathway has an independent effect on FTR rates after CRS. In future studies, FTR rates should be included in benchmarking of ERAS pathway implementation, whereas multidisciplinary (medical and surgical) perioperative management of high-risk comorbid patients with nonsurgical complications may help further reduce FTR rates after CRS.

## ACKNOWLEDMENTS

A full list of iCral Study Group Investigators is available at https://links.lww.com/DCR/C488.

## Supplementary Material

## References

[1] Silber JH Williams SV Krakauer H Schwartz JS. Hospital and patient characteristics associated with death after surgery. A study of adverse occurrence and failure to rescue. Med Care. 1992;30:615–629. 1614231 10.1097/00005650-199207000-00004

[2] Rosero EB Modrall JG Joshi GP. Failure to rescue after major abdominal surgery: the role of hospital safety net burden. Am J Surg. 2020;220:1023–1030. 32199603 10.1016/j.amjsurg.2020.03.014

[3] Portuondo JI Shah SR Singh H Massarweh NN. Failure to rescue as a surgical quality indicator: current concepts and future directions for improving surgical outcomes. Anesthesiology. 2019;131:426–437. 30860985 10.1097/ALN.0000000000002602

[4] Wells CI Bhat S Xu W. Variation in the definition of ‘failure to rescue’ from postoperative complications: a systematic review and recommendations for outcome reporting. Surgery. 2024;175:1103–1110. 38245447 10.1016/j.surg.2023.12.006

[5] Lillo-Felipe M Ahl Hulme R Sjolin G. Hospital academic status is associated with failure-to-rescue after colorectal cancer surgery. Surgery. 2021;170:863–869. 33707039 10.1016/j.surg.2021.01.050

[6] Henneman D van Leersum NJ Ten Berge M. Failure-to-rescue after colorectal cancer surgery and the association with three structural hospital factors. Ann Surg Oncol. 2013;20:3370–3376. 23732859 10.1245/s10434-013-3037-z

[7] Pandit V Jehan F Zeeshan M. Failure to rescue in postoperative patients with colon cancer: time to rethink where you get surgery. J Surg Res. 2019;234:1–6. 30527459 10.1016/j.jss.2018.08.046

[8] Lillo-Felipe M Ahl Hulme R Forssten MP. Center-level procedure volume does not predict failure-to-rescue after severe complications of oncologic colon and rectal surgery. World J Surg. 2021;45:3695–3706. 34448919 10.1007/s00268-021-06296-w PMC8572842

[9] Ko A Aquino L Melo N Alban RF. Surgical outcomes and failure-to-rescue events after colectomy in teaching hospitals: a nationwide analysis. Am J Surg. 2016;212:1133–1139. 27765178 10.1016/j.amjsurg.2016.08.019

[10] Spolverato G Gennaro N Zorzi M. Failure to rescue as a source of variation in hospital mortality after rectal surgery: the Italian experience. Eur J Surg Oncol. 2019;45:1219–1224. 30904244 10.1016/j.ejso.2019.03.006

[11] Almoudaris AM Burns EM Mamidanna R. Value of failure to rescue as a marker of the standard of care following reoperation for complications after colorectal resection. Br J Surg. 2011;98:1775–1783. 22034183 10.1002/bjs.7648

[12] Wells CI Varghese C Boyle LJ. “Failure to rescue” following colorectal cancer resection: variation and improvements in a national study of postoperative mortality. Ann Surg. 2023;278:87–95. 35920564 10.1097/SLA.0000000000005650

[13] Greco M Capretti G Beretta L Gemma M Pecorelli N Braga M. Enhanced recovery program in colorectal surgery: a meta-analysis of randomized controlled trials. World J Surg. 2014;38:1531–1541. 24368573 10.1007/s00268-013-2416-8

[14] Ripollés-Melchor J Ramírez-Rodríguez JM Casans-Francés R; POWER Study Investigators Group for the Spanish Perioperative Audit and Research Network (REDGERM). Association between use of enhanced recovery after surgery protocol and postoperative complications in colorectal surgery: the postoperative outcomes within enhanced recovery after surgery protocol (POWER) study. JAMA Surg. 2019;154:725–736. 31066889 10.1001/jamasurg.2019.0995 PMC6506896

[15] Berian JR Ban KA Liu JB Ko CY Feldman LS Thacker JK. Adherence to enhanced recovery protocols in NSQIP and association with colectomy outcomes. Ann Surg. 2019;269:486–493. 29064887 10.1097/SLA.0000000000002566

[16] Ripollés-Melchor J Abad-Motos A Cecconi M; EuroPOWER Study Investigators Group. Association between use of enhanced recovery after surgery protocols and postoperative complications in colorectal surgery in Europe: the EuroPOWER international observational study. J Clin Anesth. 2022;80:110752. 35405517 10.1016/j.jclinane.2022.110752

[17] Catarci M Ruffo G Viola MG; on behalf of the Italian ColoRectal Anastomotic Leakage (iCral) study group. High adherence to enhanced recovery pathway independently reduces major morbidity and mortality rates after colorectal surgery: a reappraisal of the iCral2 and iCral3 multicenter prospective studies. Il Giornale di Chirurgia - J Italian Assoc Hospital Surg. 2023;43:e24.

[18] Catarci M Ruffo G Viola MG; Italian ColoRectal Anastomotic Leakage (iCral) study group. ERAS program adherence-institutionalization, major morbidity and anastomotic leakage after elective colorectal surgery: the iCral2 multicenter prospective study. Surg Endosc. 2022;36:3965–3984. 34519893 10.1007/s00464-021-08717-2

[19] Italian ColoRectal Anastomotic Leakage (iCral) study group. Patient-reported outcomes, return to intended oncological therapy and enhanced recovery pathways after colorectal surgery: a prospective multicenter observational investigation by the Italian ColoRectal Anastomotic Leakage (iCral 3) study group. Ann Surg Open. 2023;4:e267.

[20] von EE Altman DG Egger M. The Strengthening the Reporting of Observational Studies in Epidemiology (STROBE) statement: guidelines for reporting observational studies. J Clin Epidemiol. 2008;61:344–349. 18313558 10.1016/j.jclinepi.2007.11.008

[21] Gustafsson UO Scott MJ Hubner M. Guidelines for perioperative care in elective colorectal surgery: Enhanced Recovery After Surgery (ERAS®) Society recommendations: 2018. World J Surg. 2019;43:659–695. 30426190 10.1007/s00268-018-4844-y

[22] Ficari F Borghi F Catarci M. Enhanced recovery pathways in colorectal surgery: a consensus paper by the Associazione Chirurghi Ospedalieri Italiani (ACOI) and the PeriOperative Italian Society (POIS). G Chir. 2019;40(suppl):1–40. 32003714

[23] Clavien PA Barkun J de Oliveira ML. The Clavien-Dindo classification of surgical complications: five-year experience. Ann Surg. 2009;250:187–196. 19638912 10.1097/SLA.0b013e3181b13ca2

[24] Katayama H Kurokawa Y Nakamura K. Extended Clavien-Dindo classification of surgical complications: Japan Clinical Oncology Group postoperative complications criteria. Surg Today. 2016;46:668–685. 26289837 10.1007/s00595-015-1236-x PMC4848327

[25] Rahbari NN Weitz J Hohenberger W. Definition and grading of anastomotic leakage following anterior resection of the rectum: a proposal by the International Study Group of Rectal Cancer. Surgery. 2010;147:339–351. 20004450 10.1016/j.surg.2009.10.012

[26] KaiserMJBauerJMRamschC; MNA-International Group. Validation of the Mini Nutritional Assessment short-form (MNA-SF): a practical tool for identification of nutritional status. J Nutr Health Aging. 2009;13:782–788.19812868 10.1007/s12603-009-0214-7

[27] Kim JH. Multicollinearity and misleading statistical results. Korean J Anesthesiol. 2019;72:558–569. 31304696 10.4097/kja.19087 PMC6900425

[28] ESCP Enhanced Recovery Collaborating Group. An international assessment of the adoption of enhanced recovery after surgery (ERAS®) principles across colorectal units in 2019-2020. Colorectal Dis. 2021;23:2980–2987. 34365718 10.1111/codi.15863

[29] Lafonte M Cai J Lissauer ME. Failure to rescue in the surgical patient: a review. Curr Opin Crit Care. 2019;25:706–711. 31567517 10.1097/MCC.0000000000000667

[30] Italian ColoRectal Anastomotic Leakage (iCral) Study Group. Anastomotic leakage after elective colorectal surgery: a prospective multicentre observational study on use of the Dutch leakage score, serum procalcitonin and serum C-reactive protein for diagnosis. BJS Open. 2020;4:499–507. 32134216 10.1002/bjs5.50269 PMC7260403

[31] Borghi F Migliore M Cianflocca D; Italian ColoRectal Anastomotic Leakage (iCral) study group. Management and 1-year outcomes of anastomotic leakage after elective colorectal surgery. Int J Colorectal Dis. 2021;36:929–939. 33118101 10.1007/s00384-020-03777-7

[32] Augestad KM Skyrud KD Lindahl AK Helgeland J. Hospital variations in failure to rescue after abdominal surgery: a nationwide, retrospective observational study. BMJ Open. 2023;13:e075018. 10.1136/bmjopen-2023-075018 PMC10661059 37977874

[33] Horta AB Geraldes C Salgado C Vieira S Xavier M Papoila AL. A multivariable prediction model to select colorectal surgical patients for co-management. Acta Med Port. 2021;34:118–127. 33164728 10.20344/amp.12996

[34] Gustafsson UO Hausel J Thorell A Ljungqvist O Soop M Nygren J; Enhanced Recovery After Surgery Study Group. Adherence to the enhanced recovery after surgery protocol and outcomes after colorectal cancer surgery. Arch Surg. 2011;146:571–577. 21242424 10.1001/archsurg.2010.309

[35] Diers J Baum P Matthes H Germer CT Wiegering A. Mortality and complication management after surgery for colorectal cancer depending on the DKG minimum amounts for hospital volume. Eur J Surg Oncol. 2021;47:850–857. 33020007 10.1016/j.ejso.2020.09.024

[36] Localio AR Berlin JA Ten Have TR Kimmel SE. Adjustments for center in multicenter studies: an overview. Ann Intern Med. 2001;135:112–123. 11453711 10.7326/0003-4819-135-2-200107170-00012

