# Supplementary material for: Enhanced Recovery Independently Lowers Failure to Rescue After Colorectal Surgery
Source: Dis Colon Rectum. 2025 Feb 11;68(5):616–26. doi: 10.1097/DCR.0000000000003655 (PMC11999097; doi:10.1097/DCR.0000000000003655)
Supplement: Supplementary file 1 [file dcr-68-0616-s002.pdf]

Supplemental Table 1: Inclusion and exclusion criteria shared by the two studies.

Inclusion criteria	• Patients undergoing colo-rectal resection with anastomosis (laparoscopic, robotic, open or converted approach), including Hartmann's reversals<br>• American Society of Anesthesiologists (ASA) class I, II or III<br>• Elective or delayed urgency (> 24-48 hours from admission) surgery<br>• Patient's written informed consent for inclusion in the study and processing of sensitive data
Exclusion criteria	• Pregnancy<br>• Hyperthermic chemotherapy (HIPEC) for carcinomatosis<br>• Incomplete data

Supplemental Table 2: Definition of adherence to ERAS pathway items shared by the two studies.

ITEM	Adherence criteria
Prehabilitation	All patients showing MNA-SF < 12 (malnourished or suspect for malnutrition) and BMI > 30 (obesity) receive specific nutritional consultation. Patient receives a standard protocol of physical activity to be accomplished in the preoperative period. Patient and his familiars/caregivers are screened for anxiety/depression concerning diagnosis and related procedure; if present, psychologic consultation is warranted
Counseling	Patient and his familiars/caregivers receive full information and suggestions regarding perioperative program from surgeon, anesthesiologist and case-manager
Preoperative immunonutrition	Patient is administered Impact Oral ™ (Nestlè Health Science, Italy) 330 ml per os, three briks per day during 5 days or two bricks per day during 7 days preceding surgery
Antithrombotic prophylaxis	Patient receives graduate compression stockings and/or pneumatic compression device, together with prophylaxis with low molecular weight heparin during the perioperative period, to be extended up to 28 days after surgery in case of malignancy
Antibiotic prophylaxis	Patient is administered i.v. antibiotic 30 to 60 minutes before incision, according to local protocols
No mechanical bowel preparation	No routine mechanical bowel preparation is used, except in case of anticipated need for covering stoma
Oral carbohydrates load & preoperative fasting	Carbohydrates rich beverage (12.5% maltrodextrins, PreOp™, Nutricia Italy) is given preoperatively (800 ml on the evening before surgery and 400 ml 2 to 3 hours before surgery). Preoperative fasting is limited to two hours for clear liquids (water, coffee, tea) and to 6 hours for milk and solid food
No premedication	No long- or medium-action sedatives. Short and ultra-short acting sedatives (e.g. Lorazepam, Midazolam, Methohexital, Dexmedetomidine, Ketamine) are allowed before performing spinal, epidural or loco-regional anesthesia
PONV prophylaxis	Postoperative nausea/vomiting (PONV) prophylaxis is administered according to individual risk assessment (Apfel score) through a multimodal approach
Normothermia	Body temperature is monitored during surgery, utilizing fluid warmers and/or thermic blankets as necessary
Standard anesthesia protocol	General anesthesia through short-acting anesthetics, cerebral activity monitoring to enhance recovery and to reduce postoperative delirium, anesthesia level monitoring and complete reversal of neuromuscular blockade
Fluid management	Intraoperative restrictive fluid therapy (defined as maintenance fluids at <2 ml/kg/h) or goal-oriented fluid therapy (stroke volume)
Multimodal analgesia	Use of more than two drugs or analgesia strategies (TAP-block or spinal anesthesia for minimally invasive surgery; thoracic epidural anesthesia for open surgery) in order to reduce the use of opiates
Minimally invasive surgery	Patient submitted to laparoscopic, robotic or video-assisted surgery (conversions to open surgery included on a intention-to-treat basis)
No nasogastric tube	Nasogastric tube, if used, is removed at the end of surgery
No drain	No drain is placed in the abdominal cavity (pelvic drain allowed for pelvic surgery with low colorectal anastomosis)
Bladdder catheter	Urinary catheter removed on POD 1 (up to POD 2 in case of pelvic surgery)
Early mobilization	Patient receives passive mobilization on POD 0, active mobilization on POD 1
Early oral feeding	Patient receives liquid oral diet starting 6 hours after surgery and semisolid diet starting on POD 1
Pre-discharge check	Patient is checked just before discharge at home concerning adequate oral intake, bowel function, adequate pain control, active mobilization, no clinical/serological evidence of any postoperative complication, full agreement to go home
