# Supplementary material for: Enhanced Recovery Independently Lowers Failure to Rescue After Colorectal Surgery
Source: Dis Colon Rectum. 2025 Feb 11;68(5):616–26. doi: 10.1097/DCR.0000000000003655 (PMC11999097; doi:10.1097/DCR.0000000000003655)
Supplement: Supplementary file 2 [file dcr-68-0616-s003.pdf]

ACKNOWLEDGMENTS

iCral Study Group Investigators

Paolo Ciano, M.D., General Surgery Unit, Sandro Pertini Hospital, ASL Roma 2, Italy; Michele Benedetti, M.D., General Surgery Unit, Sandro Pertini Hospital, ASL Roma 2, Italy; Leonardo Antonio Montemurro, M.D., General Surgery Unit, Sandro Pertini Hospital, ASL Roma 2, Italy; Maria Sole Mattei, M.D., General Surgery Unit, Sandro Pertini Hospital, ASL Roma 2, Italy; Elena Belloni, M.D., General Surgery Unit, Sandro Pertini Hospital, ASL Roma 2, Italy; Matteo Di Carlo, M.D., General Surgery Unit, Sandro Pertini Hospital, ASL Roma 2, Italy; Elisa Bertocchi, M.D., General Surgery Unit, IRCCS Sacro Cuore Don Calabria Hospital, Negrar di Valpolicella (VR), Italy; Gaia Masini, M.D., General Surgery Unit, IRCCS Sacro Cuore Don Calabria Hospital, Negrar di Valpolicella (VR), Italy; Amedeo Altamura, M.D., General Surgery Unit, Cardinale G. Panico Hospital, Tricase (LE), Italy; Francesco Rubichi, M.D., General Surgery Unit, Cardinale G. Panico Hospital, Tricase (LE), Italy; Daniele Parlanti, M.D., General Surgery Unit, Infermi Hospital, Rimini, Italy; Gabriele Vago, M.D., General Surgery Unit, Infermi Hospital, Rimini, Italy; Carlo Di Marco, M.D., General Surgery Unit, AULSS2 Marca Trevigiana, Conegliano Veneto (TV), Italy; Lorenzo Pandolfini, M.D., General Surgery Unit, Santa Maria Annunziata & Serristori Hospital, Florence, Italy; Alessandro Falsetto, M.D., General Surgery Unit, Santa Maria Annunziata & Serristori Hospital, Florence, Italy; Antonio Ferronetti, M.D., General & Oncologic Surgery Unit, Evangelico Betania Hospital, Napoli, Italy; Marco Clementi, M.D., General Surgery Unit, University Hospital, L'Aquila, Italy; Ferdinando Ficari, M.D., General Surgery and IBD Unit, Careggi University Hospital, Firenze, Italy; Francesco Giudici, M.D., General Surgery and IBD Unit, Careggi University Hospital, Firenze, Italy; Fabio Cianchi, M.D., General Surgery and IBD Unit, Careggi University Hospital, Firenze, Italy; Paolo Delrio, M.D., Colorectal Surgical Oncology, Istituto Nazionale per lo Studio e la Cura dei Tumori, "Fondazione Giovanni Pascale IRCCS-Italia", Napoli, Italy; Ugo Pace, M.D., Colorectal Surgical Oncology, Istituto Nazionale per lo Studio e la Cura dei Tumori, "Fondazione Giovanni Pascale IRCCS-Italia", Napoli, Italy; Felice Pirozzi, M.D., General Surgery Unit, ASL Napoli 2 Nord, Pozzuoli (NA), Italy; Antonio Sciuto, M.D., General Surgery Unit, ASL Napoli 2 Nord, Pozzuoli (NA), Italy; Felice Borghi, M.D., General & Oncologic Surgery Unit, Department of Surgery, Santa Croce e Carle Hospital, Cuneo, Italy; Desirée Cianflocca, M.D., General & Oncologic Surgery Unit, Department of Surgery, Santa Croce e Carle Hospital, Cuneo, Italy; Marco Migliore, M.D., General & Oncologic Surgery Unit, Department of Surgery, Santa Croce e Carle Hospital, Cuneo, Italy; Raffaele De Luca, M.D., Department of Surgical Oncology, IRCCS Istituto Tumori "Giovanni Paolo II", Bari, Italy; Michele Simone, M.D., Department of Surgical Oncology, IRCCS Istituto Tumori "Giovanni Paolo II", Bari, Italy; Alberto Patriti, M.D., Department of Surgery, Marche Nord Hospital, Pesaro e Fano (PU), Italy; Valerio Sisti, M.D., Department of Surgery, Marche Nord Hospital, Pesaro e Fano (PU), Italy; Marcella Lodovica Ricci, M.D., Department of Surgery, Marche Nord Hospital, Pesaro e Fano (PU), Italy; Walter Siquini, M.D., General Surgery Unit, S. Lucia Hospital, Macerata, Italy; Alessandro Cardinali, M.D., General Surgery Unit, S. Lucia Hospital, Macerata, Italy; Stefano D'Ugo, M.D., General Surgery Unit, "V. Fazzi" Hospital, Lecce, Italy; Marcello Spampinato, M.D., General Surgery Unit, "V. Fazzi" Hospital, Lecce, Italy; Stefano Scabini, M.D., General & Oncologic Surgery Unit, IRCCS "San Martino" National Cancer Center, Genova, Italy; Alessandra Aprile, M.D., General & Oncologic Surgery Unit, IRCCS

“San Martino” National Cancer Center, Genova, Italy; Domenico Soriero, M.D., General & Oncologic Surgery Unit, IRCCS “San Martino” National Cancer Center, Genova, Italy; Marco Caricato, M.D., F.A.C.S., Colorectal Surgery Unit, Policlinico Campus BioMedico, Roma, Italy; Gabriella Teresa Capolupo, M.D., F.A.C.S., Colorectal Surgery Unit, Policlinico Campus BioMedico, Roma, Italy; Giusto Pignata, M.D., 2nd General Surgery Unit 2, Spedali Civili di Brescia, Italy; Jacopo Andreuccetti, M.D., 2nd General Surgery Unit 2, Spedali Civili di Brescia, Italy; Ilaria Canfora, M.D., 2nd General Surgery Unit 2, Spedali Civili di Brescia, Italy; Andrea Liverani, M.D., General Surgery Unit, Regina Apostolorum Hospital, Albano Laziale (RM), Italy; Giuseppe Lamacchia, M.D., General Surgery Unit, Regina Apostolorum Hospital, Albano Laziale (RM), Italy; Claudia Franceschilli, M.D., General Surgery Unit, Regina Apostolorum Hospital, Albano Laziale (RM), Italy; Roberto Campagnacci, M.D., General Surgery Unit, “C. Urbani” Hospital, Jesi (AN), Italy; Angela Maurizi, M.D., General Surgery Unit, “C. Urbani” Hospital, Jesi (AN), Italy; Pierluigi Marini, M.D., General & Emergency Surgery Unit, San Camillo-Forlanini Hospital, Roma, Italy; Grazia Maria Attinà, M.D., General & Emergency Surgery Unit, San Camillo-Forlanini Hospital, Roma, Italy; Ugo Elmore, M.D., Gastroenterologic Surgery Unit, IRCCS S. Raffaele Hospital, Milano, Italy; Francesco Puccetti, M.D., Gastroenterologic Surgery Unit, IRCCS S. Raffaele Hospital, Milano, Italy; Francesco Corcione, M.D., General Oncologic and Mininvasive Surgery Unit, “Federico II” University, Napoli, Italy; Umberto Bracale, M.D., General Oncologic and Mininvasive Surgery Unit, “Federico II” University, Napoli, Italy; Roberto Peltrini, M.D., General Oncologic and Mininvasive Surgery Unit, “Federico II” University, Napoli, Italy; Maria Michela Di Nuzzo, M.D., General Oncologic and Mininvasive Surgery Unit, “Federico II” University, Napoli, Italy; Roberto Santoro, M.D., General Oncologic Surgery Unit, Belcolle Hospital, Viterbo, Italy; Pietro Amodio, M.D., General Oncologic Surgery Unit, Belcolle Hospital, Viterbo, Italy; Massimo Carlini, M.D., F.A.C.S., General Surgery Unit, S. Eugenio Hospital, ASL Roma 2, Italy; Domenico Spoletini, M.D., General Surgery Unit, S. Eugenio Hospital, ASL Roma 2, Italy; Rosa Marcellinaro, M.D., General Surgery Unit, S. Eugenio Hospital, ASL Roma 2, Italy; Antonio Giuliani, M.D., General Surgery Unit, S. Carlo Hospital, Potenza, Italy; Giovanni Del Vecchio, M.D., General Surgery Unit, S. Carlo Hospital, Potenza, Italy; Mario Sorrentino, M.D., General Surgery Unit, Latisana-Palmanova Hospital, Friuli Centrale University (UD); Massimo Stefanoni, M.D., General Surgery Unit, Latisana-Palmanova Hospital, Friuli Centrale University (UD); Giovanni Ferrari, M.D., General Oncologic and Mininvasive Surgery Unit, Great Metropolitan Niguarda Hospital, Milano, Italy; Carmelo Magistro, M.D., General Oncologic and Mininvasive Surgery Unit, Great Metropolitan Niguarda Hospital, Milano, Italy; Gianandrea Baldazzi, MD, General Surgery Unit, ASST Nord Milano, Sesto San Giovanni (MI), Italy; Diletta Cassini, M.D., General Surgery Unit, ASST Nord Milano, Sesto San Giovanni (MI), Italy; Alberto Di Leo, M.D., General and Minimally Invasive Surgery Unit, San Camillo Hospital, Trento, Italy; Lorenzo Crepaz, M.D., General and Minimally Invasive Surgery Unit, San Camillo Hospital, Trento, Italy; Augusto Verzelli, M.D., General Surgery Unit, Profili Hospital, Fabriano (AN), Italy; Andrea Budassi, M.D., General Surgery Unit, Profili Hospital, Fabriano (AN), Italy; Giuseppe Sica, M.D., Minimally Invasive Surgery Unit, Policlinico Tor Vergata University Hospital, Roma, Italy; Bruno Sensi, M.D., Minimally Invasive Surgery Unit, Policlinico Tor Vergata University Hospital, Roma, Italy; Stefano Rausei, M.D., General Surgery Unit, Gallarate Hospital (VA), Gallarate, Italy; Silvia Tenconi, M.D., General Surgery Unit, Gallarate Hospital (VA), Gallarate, Italy; Davide Cavaliere, M.D., General & Oncologic Surgery Unit, AUSL Romagna, Forlì (FC), Italy; Leonardo Solaini, M.D., General & Oncologic

Surgery Unit, AUSL Romagna, Forlì (FC), Italy; Giorgio Ercolani, M.D., General & Oncologic Surgery Unit, AUSL Romagna, Forlì (FC), Italy; Gian Luca Baiocchi, M.D., F.A.C.S., General Surgery Unit 3, Department of Clinical and Experimental Sciences, University of Brescia, Brescia, Italy; Sarah Molfino, M.D., General Surgery Unit 3, Department of Clinical and Experimental Sciences, University of Brescia, Brescia, Italy; Marco Milone, M.D., General & Endoscopic Surgery Unit, "Federico II" University, Napoli, Italy; Giovanni Domenico De Palma, M.D., General & Endoscopic Surgery Unit, "Federico II" University, Napoli, Italy; Giovanni Ciaccio, M.D., General Surgery Unit, S. Elia Hospital, Caltanissetta, Italy; Paolo Locurto, M.D., General Surgery Unit, S. Elia Hospital, Caltanissetta, Italy; Giovanni Tebala, M.D., General Surgery Unit, S. Maria Hospital, Terni, Italy; Antonio Di Cintio, M.D., General Surgery Unit, S. Maria Hospital, Terni, Italy; Luigi Boni, M.D., F.A.C.S., General Surgery Unit, Fondazione IRCCS Ca' Granda, Policlinico Maggiore Hospital, Milano, Italy; Elisa Cassinotti, M.D., General Surgery Unit, Fondazione IRCCS Ca' Granda, Policlinico Maggiore Hospital, Milano, Italy; Stefano Mancini, M.D., General & Oncologic Surgery Unit, San Filippo Neri Hospital, ASL Roma 1, Italy; Andrea Sagnotta, M.D., Ph.D., General & Oncologic Surgery Unit, San Filippo Neri Hospital, ASL Roma 1, Italy; Mario Guerrieri, M.D., Surgical Clinic, Torrette Hospital, University of Ancona, Ancona, Italy; Monica Ortenzi, M.D., Surgical Clinic, Torrette Hospital, University of Ancona, Ancona, Italy; Roberto Persiani, M.D., General Surgery Unit, Fondazione Policlinico Universitario Agostino Gemelli IRCCS, Roma, Italy; Alberto Biondi, M.D., General Surgery Unit, Fondazione Policlinico Universitario Agostino Gemelli IRCCS, Roma, Italy; Andrea Lucchi, M.D., F.A.C.S., General Surgery Unit, "Ceccarini" Hospital, Riccione (RN), Italy; Giacomo Martorelli, M.D., General Surgery Unit, "Ceccarini" Hospital, Riccione (RN), Italy; Dario Parini, M.D., General Surgery Unit, S. Maria della Misericordia Hospital, Rovigo, Italy; Maurizio De Luca, M.D., General Surgery Unit, S. Maria della Misericordia Hospital, Rovigo, Italy; Antonino Spinelli, M.D., Colorectal Surgery Unit, Humanitas University, Rozzano (MI), Italy; Francesco Carrano, M.D., Colorectal Surgery Unit, Humanitas University, Rozzano (MI), Italy; Michele Genna, M.D., General & Bariatric Surgery Unit, University Hospital, Verona, Italy; Francesca Fior, M.D., General & Bariatric Surgery Unit, University Hospital, Verona, Italy; Andrea Coratti, M.D., General Surgery Unit, Misericordia Hospital, Grosseto, Italy; Giuseppe Giuliani, M.D., General Surgery Unit, Misericordia Hospital, Grosseto, Italy; Dario Scala, M.D., Abdominal Oncologic Surgery Unit, Basilicata Oncologic Hospital, Rionero in Vulture (PZ), Italy; Graziella Marino, M.D., Abdominal Oncologic Surgery Unit, Basilicata Oncologic Hospital, Rionero in Vulture (PZ), Italy; Andrea Muratore, M.D., General Surgery Unit, "E. Agnelli" Hospital, Pinerolo (TO), Italy; Patrizia Marsanic, M.D., General Surgery Unit, "E. Agnelli" Hospital, Pinerolo (TO), Italy; Umberto Rivolta, M.D., General Surgery Unit, Fornaroli Hospital, ASST Milano Ovest, Magenta (MI), Italy; Giuliano Sarro, M.D., General Surgery Unit, Fornaroli Hospital, ASST Milano Ovest, Magenta (MI), Italy; Micaela Piccoli, M.D., General Surgery Unit, Civil Hospital, Baggiovara (MO), Italy; Francesca Pecchini, M.D., General Surgery Unit, Civil Hospital, Baggiovara (MO), Italy; Carlo Talarico, M.D., General Surgery Unit, Villa dei Gerani Hospital, Vibo Valentia (VV), Italy; Vincenzo Greco, M.D., General Surgery Unit, Villa dei Gerani Hospital, Vibo Valentia (VV), Italy; Alessandro Carrara, M.D., 2nd General Surgery Unit, S. Chiara Hospital, Trento, Italy; Michele Motter, M.D., 2nd General Surgery Unit, S. Chiara Hospital, Trento, Italy; Giuseppe Tirone, M.D., 2nd General Surgery Unit, S. Chiara Hospital, Trento, Italy; Mauro Totis, M.D., Colorectal Surgery Unit, San Gerardo Hospital, ASST Monza, Italy; Nicolò Tamini, M.D., Colorectal Surgery Unit, San Gerardo Hospital, ASST Monza, Italy;

Marco Braga, M.D., Colorectal Surgery Unit, San Gerardo Hospital, ASST Monza, Italy; Franco Roviello, M.D., Surgical Clinic, University of Siena, Siena, Italy; Roberto Piagnerelli, M.D., Surgical Clinic, University of Siena, Siena, Italy; Alessandro Anastasi, M.D., General Surgery Unit, San Giovanni di Dio Hospital, Firenze, Italy; Giuseppe Canonico, M.D., General Surgery Unit, San Giovanni di Dio Hospital, Firenze, Italy; Gianluca Guercioni, M.D., General Surgery Unit, "C. e G. Mazzoni" Hospital, Ascoli Piceno, Italy; Simone Cicconi, M.D., General Surgery Unit, "C. e G. Mazzoni" Hospital, Ascoli Piceno, Italy; Irene Marziali, M.D., General Surgery Unit, "C. e G. Mazzoni" Hospital, Ascoli Piceno, Italy; Giuseppe Maria Ettorre, M.D., General & Transplant Surgery Unit, San Camillo-Forlanini Hospital, Roma, Italy; Marco Colasanti, M.D., General & Transplant Surgery Unit, San Camillo-Forlanini Hospital, Roma, Italy; Mauro Montuori, M.D., General & Mininvasive Surgery Unit, S. Pietro Hospital, Ponte San Pietro (BG), Italy; Enrico Pinotti, M.D., General & Mininvasive Surgery Unit, S. Pietro Hospital, Ponte San Pietro (BG), Italy; Pierpaolo Mariani, M.D., General Surgery Unit, Pesenti Fenaroli Hospital, Alzano Lombardo (BG), Italy; Roberta Carminati, M.D., General Surgery Unit, Pesenti Fenaroli Hospital, Alzano Lombardo (BG), Italy; Nicolò de Manzini, M.D., Surgical Clinic, University of Trieste, Trieste, Italy; Edoardo Osenda, M.D., Surgical Clinic, University of Trieste, Trieste, Italy; Annibale Donini, M.D., General & Emergency Surgery Unit, University of Perugia, Perugia, Italy; Luigina Graziosi, M.D., General & Emergency Surgery Unit, University of Perugia, Perugia, Italy; Mariano Fortunato Armellino, M.D., General & Emergency Surgery Unit, S. Giovanni di Dio e Ruggi d'Aragona Hospital, Salerno, Italy; Ciro De Martino, M.D., General & Emergency Surgery Unit, S. Giovanni di Dio e Ruggi d'Aragona Hospital, Salerno, Italy; Lucio Taglietti, M.D., General Surgery Unit, ASST Valcamonica, Esine (BS), Italy; Silvia Ruggiero, M.D., General Surgery Unit, ASST Valcamonica, Esine (BS), Italy; Gabriele Anania, M.D., General & Laparoscopic Surgery Unit, University Hospital, Ferrara, Italy; Matteo Chiozza, M.D., General & Laparoscopic Surgery Unit, University Hospital, Ferrara, Italy; Mariantonietta Di Cosmo, M.D., General & Upper GI Surgery Unit, University Hospital, Verona, Italy; Daniele Zigiotto, M.D., General & Upper GI Surgery Unit, University Hospital, Verona, Italy; Carlo Vittorio Feo, M.D., General Surgery Unit, Delta Hospital, Lagosanto (FE), Italy; Fioralba Pindozzi, M.D., General Surgery Unit, Delta Hospital, Lagosanto (FE), Italy; Paolo Millo, M.D., General Surgery Unit, "U. Parini" Regional Hospital, Aosta, Italy; Manuela Grivon, M.D., General Surgery Unit, "U. Parini" Regional Hospital, Aosta, Italy; Corrado Pedrazzani, M.D., General & HPB Surgery Unit, University Hospital, Verona, Italy; Cristian Conti, M.D., General & HPB Surgery Unit, University Hospital, Verona, Italy; Silvio Guerriero, M.D., General Surgery Unit, "F. Murri" Hospital, Fermo, Italy; Lorenzo Organetti, M.D., General Surgery Unit, "F. Murri" Hospital, Fermo, Italy; Andrea Costanzi, M.D., General Surgery Unit, S. Leopoldo Hospital, Merate (LC), Italy; Michela Monteleone, M.D., General Surgery Unit, S. Leopoldo Hospital, Merate (LC), Italy; Nereo Vettoretto, M.D., General Surgery Unit, Spedali Civili of Brescia, Montichiari (BS), Italy; Emanuele Botteri, M.D., General Surgery Unit, Spedali Civili of Brescia, Montichiari (BS), Italy; Federico Marchesi, M.D., Surgical Clinic, University of Parma, Parma, Italy; Giorgio Dal Monte, M.D., Surgical Clinic, University of Parma, Parma, Italy; Massimo Basti, M.D., General Surgery Unit, Spirito Santo Hospital, Pescara, Italy; Diletta Frazzini, M.D., General Surgery Unit, Spirito Santo Hospital, Pescara, Italy; Graziano Longo, M.D., General Surgery Unit, Policlinico Casilino, Roma, Italy; Simone Santoni, M.D., General Surgery Unit, Policlinico Casilino, Roma, Italy; Moreno Cicetti, M.D., General Surgery Unit, S. Maria della Misericordia Hospital, Urbino (PU), Italy; Gabriele La Gioia, M.D., General Surgery Unit, S. Maria della Misericordia Hospital,

Urbino (PU), Italy; Giuseppe Brisinda, M.D., General Surgery Unit, San Giovanni di Dio Hospital, Crotone, Italy; Maria Michela Chiarello, M.D., General Surgery Unit, San Giovanni di Dio Hospital, Crotone, Italy; Maria Cariati, M.D., General Surgery Unit, San Giovanni di Dio Hospital, Crotone, Italy; Stefano Berti, M.D., General Surgery Unit, ASL 5 Liguria POLL, La Spezia, Italy; Andrea Gennai, M.D., General Surgery Unit, ASL 5 Liguria POLL, La Spezia, Italy.
